# Improvement project to reduce surgical site infections: a retrospective cohort study

**DOI:** 10.1590/0100-6991e-20233380-en

**Published:** 2023-09-22

**Authors:** LAURA FERREIRA DIAS XAVIER, AMANDA SILVA MEDEIROS, MARIA CLARA DE SOUSA FARIAS MELO, RAPHAEL NEPOMUCENO GALVÃO SANTOS, ZENEWTON ANDRÉ DA SILVA GAMA, MARISE REIS DE FREITAS

**Affiliations:** 1 - Universidade Federal do Rio Grande do Norte, Centro de Ciências da Saúde - Natal - RN - Brasil; 2 - Universidade Federal do Rio Grande do Norte, Mestrado Profissional em Ensino na Saúde - Natal - RN - Brasil; 3 - Universidade Federal do Rio Grande do Norte, Departamento de Saúde Coletiva - Natal - RN - Brasil; 4 - Universidade Federal do Rio Grande do Norte, Departamento de Infectologia - Natal - RN - Brasil

**Keywords:** Surgical Wound Infection, Bundle, Kidney Transplantation, Infecção da Ferida Cirúrgica, Pacotes de Assistência ao Paciente, Transplante de Rim

## Abstract

**Introduction:**

Surgical site infections are one of the main problems related to health care. In Brazil, they are responsible for 14 to 16% of infections related to health care. This study sought to analyze the effect of implementing a package of measures to reduce surgical site infections (SSI) in heart surgeries, kidney transplants and herniorrhaphies and to evaluate adherence to the safe surgery checklist in a university hospital.

**Methods:**

this is a retrospective cohort study with data collection in a time series for the period from 2018 to 2020.

**Results:**

we analyzed 222 medical records referring to the surgeries under study performed in the year 2020, in which data were collected from the patients and the care package prevention measures. SSI data and adherence to the safe surgery checklist were analyzed in the years 2018, 2019 and 2020, totaling 268, 300 and 222 procedures analyzed, respectively.

**Conclusion:**

the study showed a significant reduction in the SSI rate with greater adherence to the protocol, which was not maintained and was influenced by the COVID-19 pandemic. Thus, the sustainability of this action represents a challenge to be overcome, in order to establish a safer environment for the patient and a better quality of service.

## RESUMO

**Introdução:**

as infecções do sítio cirúrgico são um dos principais agravos relacionados à assistência à saúde. No Brasil, são responsáveis por 14 a 16% das infecções relacionadas à assistência à saúde. Esse estudo buscou analisar o efeito da implementação de um pacote de medidas para redução de infecções de sítio cirúrgico (ISC) em cirurgias cardíacas, transplantes renais e herniorrafias e avaliar adesão ao checklist de cirurgia segura em um hospital universitário.

**Métodos:**

trata-se de um estudo de coorte retrospectivo com coleta de dados em série temporal relativo ao período de 2018 a 2020.

**Resultados:**

foram analisados 222 prontuários referentes às cirurgias em estudo realizadas no ano de 2020, nos quais foram coletados dados dos pacientes e do pacote de medidas de prevenção. Dados de ISC e adesão ao checklist de cirurgia segura foram analisados nos anos de 2018, 2019 e 2020, totalizando 268, 300 e 222 procedimentos analisados, respectivamente.

**Conclusão:**

o estudo evidenciou uma redução significativa da taxa de ISC com a maior adesão ao protocolo, a qual não foi mantida e sofreu influência da pandemia de COVID-19. Assim, a sustentabilidade dessa ação representa um desafio a ser contornado, a fim de estabelecer um ambiente mais seguro para o paciente e uma melhor qualidade do serviço.

## INTRODUCTION

Health care-associated infections (HAIs) are adverse events resulting from a care process in a hospital or other type of health unit, representing a major challenge for health systems worldwide^1^. Among HAIs, we highlight Surgical Site Infection (SSI), a complication that occurs at the site of the operation within the first 30 days after the surgical procedure, or up to 90 days in case of prosthesis placement^2^.

SSI has a multifactorial cause and is closely related to the hospital environment and its professional team. Although avoidable, it is one of the main risks to patient safety in health services in Brazil, considered a significant predictor of death, with a risk of mortality from 2 to 11 times higher when compared with cases without infection. Likewise, it is associated with a longer hospital stay, an average of 7 to 11 days. Therefore, it results in higher medical care costs and higher readmission rates, in addition to a negative impact on patients’ quality of life^2^ ^-^ ^4^.

In low- and middle-income countries, 11% of patients undergoing surgery are infected in the process, this being the most frequent type of infection acquired during the provision of health services^1^. In the United States, this index is 2.6%, and in European countries, 1.6%^5^. In Brazil, SSI ranks third among HAIs, comprising 14% to 16% of those^6^.

Clean surgeries performed on sterile tissue in the absence of a local infectious or inflammatory process or gross technical failures have low infection rates, from 1% to 5%, thus representing an important indicator of quality in surgery^4^ ^,^ ^7^. Among them are cardiac surgeries and herniorrhaphies. In developed countries, the rate of SSI in cardiac surgery ranges from 0.5% to 10%^8^ ^,^ ^9^. In developing ones, such as Brazil, this figure can vary from 3.5% to 21.0%, while herniorrhaphies display a value close to that of developed countries, with 4.1%^10^ ^,^ ^11^.

Potentially contaminated surgeries, on their turn, include those in which there is handling of tissues colonized by a small number of microbiota, or in colonized tissues absent of an infectious, inflammatory process, or technical failures, such as urinary tract surgeries, like kidney transplantation. The rate of SSI in kidney transplants ranges from 4.0% to 7.5%^8^ ^,^ ^9^. In Brazil, second in kidney transplants in the world, the rate of SSI in these surgeries is around 13.6%^12^.

In the hospital where the study was carried out, SSI has been the most prevalent infection in the last three years, according to the Health Care-Related Infections Control Service (SCIRAS). In the period from June 2017 to July 2018, infection rates in clean surgeries such as cardiac and hernia surgery were 19% and 7%, respectively, and in kidney transplant surgery, 18.7% (SCIRAS - unpublished data). These data encouraged the development of the REDISC (Reduction of Surgical Site Infection) project, a multifaceted improvement project started in June 2019 at the university hospital, which groups together initiatives related to the prevention of surgical site infection, based on the measures recommended by the World Health Organization - WHO. Such measures were gathered in a set of practices that, together, result in improvement^13^.

The project was designed to implement improvement cycles with the Plan, Do, Study and Act (PDSA) model, used to systematize the change process - planning it, experimenting with it, observing the results, and acting in accordance with what was learned^14^. The tested and implemented preventive measures were preoperative bath, adequate shaving, adequate antibiotic prophylaxis, maintenance of normoglycemia, and perioperative normothermia (Chart 1).

**Chart 1:** Definition and analysis criteria of the elements of the Surgical Site Infection prevention program.

Variables	Compliance criteria
Antibiotic prophylaxis*	Kidney transplantation: cefazolin Cardiac surgeries with or without CPB: cefazolin or cefuroxime Herniorrhaphy: cefazolin **All antimicrobials must be administered with an interval of 30 minutes to 1 hour before the surgical incision, ***Vancomycin is the antimicrobial of choice for patients colonized by methicillin-resistant Staphylococcus aureus and should be administered 2 hours before the surgical incision. ****Intraoperative dose interval: 4/4h for cefazolin and cefuroxime.
Trichotomy	Avoid trichotomy. When necessary, perform it up to 2 hours before surgery with the electric clipper in the operating room.
Perioperative bath (heart and kidney transplants)*****	Perform a bath with 2% degerming chlorhexidine the night before the procedure and up to 2 hours before.
Banho perioperatório (herniorrafias)^&^	Direct the patient to perform a spray bath up to 2 hours before surgery.
Perioperative bath (herniorrhaphies)^&^	Maintain the patient's temperature above 35.5°C in the peri, intra, and postoperative periods. Record in anesthetic and perioperative control sheets.
Glycemia (heart and kidney transplants)	Maintain blood glucose below 180mg/dL in the peri, intra, and postoperative period. Record in anesthetic and perioperative control sheets.

*according to the antibiotic prophylaxis protocol adopted at the institution; *****For urgent and emergency surgeries, the above protocols are not used; ^&^Hernia surgery has admission on the day of surgery; CPB: cardiopulmonary bypass.

In addition to these, the program encouraged adherence to the safe surgery checklist, a patient safety tool already implemented in the hospital since May 2016, which contributes to the reduction of adverse events related to surgical procedure, including SSI.

Thus, the present study sought to describe the effects of implementing the set of preventive measures on surgical site infection in cardiac surgeries, kidney transplants (Kidney Tx), and herniorrhaphies at the university hospital, as well as to assess the adherence to the safe surgery checklist.

## METHODS

This research is a retrospective cohort study with data collection in a time series for the period from January 2018 to December 2020. Data on adherence to the safe surgery checklist and surgical site infection comprehended the period from January to December 2019. Considering that the intervention to prevent infection took place from June 2019 on, the data related to this were obtained for the period from January to December 2020, from the analysis of medical records of patients undergoing the studied procedures. 

### Context

The study hospital has 247 beds, of which 87 are surgical, 87 are clinical, 19 are adult intensive care, and five are pediatric intensive care. The surgical center has seven general operating rooms, in addition to outpatient and ophthalmological operating rooms. The hospital performed, on average, 550 surgeries/month in the pre-pandemic period.

### Data collection

Data related to surgical site infection were provided by SCIRAS, through the SSI surveillance system, which uses the following SSI diagnostic criteria2:

1. Superficial incisional: occurs in the first 30 days after surgery and involves only the skin and subcutaneous tissue, with at least one of the following:
  - Purulent drainage from the superficial incision;
  - Positive culture of secretion or tissue from the superficial incision, obtained aseptically;
  - Superficial incision deliberately opened by the surgeon, in the presence of at least one of the following signs or symptoms: pain, increased sensitivity, local edema, hyperemia, or warmth, unless the culture is negative;
  - Diagnosis of superficial infection by the attending physician.
2. Deep incisional: occurs in the first 30 days after surgery or up to 90 days if a prosthesis is used, and involves soft tissues deep to the incision, with at least one of the following:
  - Purulent drainage from the deep incision, but not from the organ/cavity;
  - Partial or total dehiscence of the abdominal wall or opening of the wound by the surgeon, when the patient presents at least one of the following signs or symptoms: axillary temperature ≥37.8ºC, pain, or increased local sensitivity, unless the culture is negative;
  - Presence of an abscess or other evidence that the infection involves the deep layers of the wound;
  - Diagnosis of deep incisional infection by the attending physician.
3. Organ cavity: occurs within the first 30 days after surgery or up to 90 days after the procedure, if a prosthesis is used, and involves any organ or cavity that has been opened or manipulated during surgery, with at least one of the following:
  - Positive culture of aseptically obtained organ/cavity secretion or tissue;
  - Presence of an abscess or other evidence that the infection involves the deep layers of the wound;
  - Diagnosis of organ/cavity infection by the attending physician.

We collected data on patients’ demographics, comorbidities, and those related to the set of prevention measures for the period from January to December 2020, in the medical records of the patients under study, totaling 222 patients.

We organized the data in a Google Sheets spreadsheet we prepared. The following variables were collected:

1. Variables related to the patient: age, sex, hospital stay, ASA classification with graduation from I to V, comorbidities, of which the following were selected: obesity, diabetes mellitus, and autoimmune disease, in addition to smoking and use of corticosteroids or other immunosuppressants.
2. Variables related to the set of measures for SSI prevention and their respective criteria are organized in Chart 1.

We evaluated hernia surgeries only in relation to the antibiotic prophylaxis component, as the other items in the program of SSI prevention measures were not used in these procedures. The antibiotics considered incorrect are those different from the recommendation proposed by the 2018 Surgical Antibiotic Prophylaxis Protocol of the University Hospital.

Items related to adherence to the set of measures were inserted in Pareto diagrams to stratify the most common errors in adherence to the studied elements, directed to each type of surgery, with the aim of identifying and quantifying the problems.

To collect data from the safe surgery checklist, we used the same method used by SCIRAS in the previous two years (2018 and 2019). We randomly selected 17 monthly samples for the year 2020 using the Lot Quality Assurance Sampling (LQAS) method. In the condition of months with the number of procedures less than 17, we used the total number of procedures in the month, accounting for 162 procedures in 2020. The Assistance Risk Management Unit (URGA) provided the checklist data for the period from 2018 to 2019. The collected information served as a basis for verifying adherence to this checklist in the surgeries under study.

The data collected considered adherence or not to the safe surgery checklist, with adherent checklists considered those that had all 30 items completed and correct prophylactic antibiotic therapy. Procedures that did not fit one of the two mentioned criteria were considered non-adherent.

### Data analysis

To describe adherence to the set of infection prevention measures, we used descriptive statistics, by calculating the absolute and relative frequencies (percentage) of compliance with the recommended set of measures, as well as the frequencies of surgical site infections stratified by procedure or surgical specialty (herniorrhaphies, kidney transplantation, and cardiac surgeries). The analysis of the frequencies of non-compliance was also performed graphically with the aid of a Pareto Diagram, which is a bar graph representing the frequency of non-compliance with the preventive measure, plus an accumulated frequency line of non-compliances with the set of criteria.

Inferential statistical analysis was performed using trend graphs, which are time series graphs with a central reference line (median). These make it possible to identify significant variation in the indicators from a sequence of points above or below the median, as well as increasing or decreasing trends. These variations reject the null hypothesis of absence of variation with a significance level of 5%^15^.

### Ethical aspects

This research is part of a project to improve the quality of surgery at the University Hospital, which is carried out in accordance with the current rules expressed in Resolution 466/12 of the Brazilian National Health Council, under the Certificate of Presentation of Ethical Appreciation 47208321.5.0000.5292.

## RESULTS

From January 2018 to December 2020, the hospital performed, on average, 263.4 surgeries/year for herniorrhaphies, kidney transplants, and heart surgeries, with SSI medians of 5.8%, 4.4%, and 6.5% in 2018, 2019, and 2020, respectively (Table 1).

**Table 1:** Total procedures and surgical site infection (SSI), from 2018 to 2020.

	2018		2019		2020	
	n	SSI (%)	n	SSI (%)	n	SSI (%)
Total surgeries	268	5.4	300	4.7	222	10.0
Herniorraphies	142	6.3	173	1.2	85	3.5
Kidney transplants	39	12.8	48	14.6	37	10.8
Heart surgeries	87	8.0	79	24.0	100	15.0
Median SSI (%)	-	5.8	-	4.4	-	6.5

It is also worth mentioning the significant reduction in the general surgical movement in 2020 due to the COVID-19 pandemic, which resulted in the 46% reduction in the number of herniorrhaphies. However, there was an increase of about 20% in cardiac surgeries in the same period.

A retrospective analysis of patients operated on in 2020 showed that 59.5% had ASA greater than or equal to III and 12.2% had two or more comorbidities.

The length of hospital stay was 20 days for patients without infection and 39.9 days for those who developed SSI.

**Figure 1:**
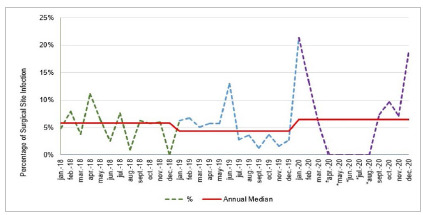
Infection trend curve in clean surgeries (hernia, kidney, and heart transplantation) from 2018 to 2020.

The SSI rate had a significant decline (p<0.05) from July 2019 on, with six consecutive points below the median, rejecting the null hypothesis of no differences (Figure 1). In 2020, the median SSI returned to the pre-intervention level.

**Figure 2:**
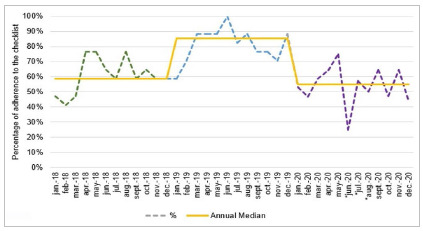
Adherence trend curve to the safe surgery checklist from 2018 to 2020. *From June to August 2020, the total number of clean surgeries was less than the monthly sample by LQAS. Source: authors, 2021.

As for adherence to the safe surgery checklist, we observed a median of 85.3% in 2019 and 55% in 2020 (Figure 2).

Complete adherence to the set of measures occurred in just two heart surgeries and in one kidney transplant, both without SSI (Table 2). Antibiotic prophylaxis and trichotomy were the most applied items, with over 80% compliance in kidney transplant surgeries and over 70% in cardiac surgeries (Table 2).

**Table 2:** Adherence to the set of measures to prevent surgical site infection in Heart Surgeries, Kidney Transplants, and Herniorrhaphies in 2020.

	Cardiac surgery	Kidney transplant	Herniorrhaphy
Element of SSI prevention measures program	Adherent procedures n (%)	Adherent procedures n (%)	Adherent procedures n (%)
Antibiotic prophylaxis	72 (72.0)	31 (83.8)	76 (89.4)
Glycemia	34 (34.0)	9 (24.3)	-
Perioperative bath	58 (58.0)	13 (35.1)	21 (24.7)
Temperature	23 (23.0)	10 (27.0)	-
Trichotomy	73 (73.0)	32 (86.5)	80 (94.1)
All	2 (2.0)	1 (2.7)	19 (22.3)
Total procedures	100	37	85

**Figure 3:**
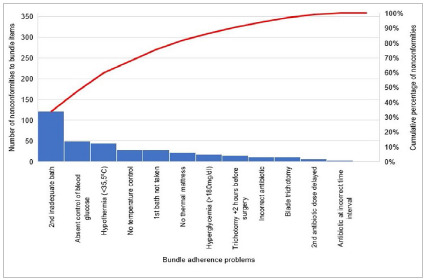
Adherence to the set of measures to prevent surgical site infection in cardiac surgeries in 2020.

According to the Pareto result, 33.8% of the problems with adherence to the set of preventive measures in cardiac surgeries in 2020 were caused by the inadequacy of the second bath, 13.7% by the non-recording or control of glycemia, and 12.3% due to intraoperative hypothermia (Figure 3).

**Figure 4:**
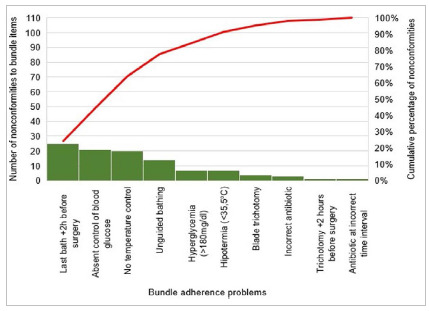
Adherence to the set of measures to prevent surgical site infection in Kidney Transplants 2020.

In kidney transplant surgeries, 24.3% of the problems were related to the period of more than two hours between the last bath and the beginning of surgery, 20.4% to unrecorded blood glucose, and 19.4% to unrecorded temperature (Figure 4).

In hernia surgeries, the prophylactic antibiotic was properly used in 89.4% of procedures. However, only 24,7% of patients received instructions regarding bathing (Table 3).

**Table 3:** Adherence to antibiotic prophylaxis in hernia surgery in 2020.

Use of prophylactic antibiotic	Total procedures	%
Correct use	76	89.4
Incorrect ATB	7	8.2
ATB out of time range	1	1.2
2nd dose of ATB outside the time interval	1	1.2
Total surgeries	85	100.0

ATB = prophylactic antibiotic.

## DISCUSSION

The present study shows that the adoption of a set of measures to prevent infection in surgery brings positive results^12^ ^,^ ^16^ ^,^ ^17^. This is corroborated by the infection indicators observed during the implementation process of the improvement project, considering that there was a significant reduction (1.4% decrease in the annual median) of SSI in 2019, with six points below the median between the months from July to December 2019. In addition, there was an increase of 26% in the annual median of adherence to the safe surgery checklist in the initial period of REDISC.

The hospital was on an upward curve of adherence to the safe surgery checklist when the set of measures of SSI prevention was implemented, in July 2019, reaching a median of 85.3% adherence that year. In 2020, there was a decline to levels below the average recorded in 2018.

Furthermore, the rate of herniorrhaphy SSI during the project period was compatible with the rates of developed countries, lower than 4.1%^11^. Also noteworthy is the good applicability of antibiotic prophylaxis, with more than 80% adherence.

The rise in the SSI index in kidney transplants and heart surgeries in 2019 may have belonged to the first half of the year, as shown in the trend curve. This pre-intervention period displayed the highest number of infected procedures compared with the other analyzed semesters.

According to the Pareto result, 33.8% of problems with adherence to the set of measures in cardiac surgeries in 2020 were caused by the inadequacy of the second bath, including in this parameter the period greater than two hours between the last bath and the beginning of surgery, the absence of guidance for bathing, or not taking it. The second most frequent problem was unrecorded blood glucose, which in the same period reached 13.7%. In third place was intraoperative hypothermia, with 12.3%. Importantly, together they correspond to an accumulated percentage of 59.8% of the problems that occurred.

In kidney transplant surgeries, the most frequent problem of adherence to the set of measures was the period of more than two hours between the last bath and the beginning of surgery, with 24.3%. The unrecorded blood glucose with 20.4% and the unrecorded temperature with 19.4% were, respectively, in the second and third places. Together, these three main causes represent an accumulated percentage of 64.1%.

There was also an increase in infection indicators in 2020, the period of the COVID-19 pandemic. In this context, the interruption of strategies resulted in a 2.1% increase in the median SSI. However, this was not repeated in other services, which observed a reduction in the overall incidence of SSI during the period in which COVID-19 containment measures were adopted, such as the use of masks and reduced movement of people^17^.

Also noteworthy is the profile of patients seen during the pandemic period, with an orientation of surgical demand for urgent situations, in view of the suspension of elective procedures, thus corroborating the increase in SSI in cardiac surgeries. Such patients have more comorbidities and a tendency to stay in the hospital for a long time, conditions that are directly related to a higher risk of infection and are reflected in the costs of treatments for patients with SSI^19^ ^,^ ^20^.

Furthermore, the non-implementation of the SSI prevention set of measures after the PDSA testing phase may reflect the impact of the health emergency imposed by COVID-19, since the quality management structure of health care and patient safety is also tested in the face of causes intrinsic to the hospital work environment, such as fear and fatigue to which professionals were subjected, or even the breakdown of leadership, with the arrival of new professionals to supply the high demand. Therefore, such situations can influence the quality of the service and put patient safety at risk^21^.

However, the literature reported antagonistic results, with the reduction of SSI associated with the increased use of personal protective equipment, suggesting the possibility of a causal relationship between the COVID-19 pandemic, the greater use of PPE, and greater adherence to perioperative care^22^.

Thus, it is necessary to encourage adherence to SSI prevention measures, in addition to other improvement projects carried out within the scope of health services, given their relevance for a positive outcome in the postoperative period. Such encouragement can be carried out through the formulation of strategies for the implementation of the set of measures by the hospital’s improvement team^23^. These strategies, in turn, include feedback to the teams and educational activities, as well as the identification and correction of problems, such as the resolution of the three most frequent problems of poor adherence to the SSI prevention set of measures in heart surgeries and kidney transplants, which can lead to an improvement of more than 60% in the adoption of the SSI reduction program actions.

The effectiveness and continuity of the improvement project is closely related to the attention of the individual and the team responsible for carrying out the proposed actions. Therefore, it is essential to monitor the SSI rates, as well as the application of the set of measures to reduce surgical site infection, to achieve results consistent with the literature and with a relevant impact on improving the service provided to patients.

### Study limitations

Inadequate recording of information in medical records and forms made it difficult to assess adherence to the SSI prevention program, making adequate data accuracy impossible. Thus, many medical records did not present information about the time and orientation for bathing, about shaving, and temperature and blood glucose measurements, and were assumed to be non-adherent, making it difficult to accurately assess the application of the analyzed elements.

## CONCLUSION

The data obtained showed that the improvement project provided a significant reduction in SSI and greater adherence to the checklist during the application phase of the PDSA cycles, in contrast to the period prior to the implementation test and during the COVID-19 pandemic.

We believe, therefore, that encouraging the incorporation and sustainability of SSI reduction projects associated with the safe surgery checklist makes an important contribution to the reduction of infections, thus providing greater service quality and patient safety. However, it is necessary to implement measures that ensure the sustainability of the recommended protocols, to observe the real impacts and areas for improvement.
